# Poly[[aqua­(μ_5_-1*H*-benzimidazole-5,6-dicarboxyl­ato)(μ_4_-1*H*-benzimidazole-5,6-dicarboxyl­ato)dibarium] 4.5-hydrate]

**DOI:** 10.1107/S160053681101590X

**Published:** 2011-05-07

**Authors:** Heng-Qiang Zhang, Xiu-Jun Zheng, Chun-Xiang Zhao, Seik Weng Ng

**Affiliations:** a Department of Chemistry, East China Normal University, Shanghai 200062, People’s Republic of China; b Department of Chemical Engineering, Huarui College, Northeast Petroleum University, Harbin 15002, People’s Republic of China; c Department of Chemistry, Zhoukou Normal University, Zhoukou 466001, People’s Republic of China; d Department of Chemistry, University of Malaya, 50603 Kuala Lumpur, Malaysia

## Abstract

The polymeric title compound, {[Ba_2_(C_9_H_4_N_2_O_4_)_2_(H_2_O)]·4.5H_2_O}_*n*_, adopts a layer structure parallel to (001) in which adjacent Ba^II^ atoms are connected by two benzimidazole-5,6-dicarboxyl­ate dianions, one functioning in a μ_4_-bridging mode and the other in a μ_5_-bridging mode. The Ba atom having water in its coordination environment as well as the Ba atom without water exist in a nine-coordinate polyhedron of O atoms; the geometry is difficult to derive. Lattice water mol­ecules occupy the space between layers and inter­act with the layers through O—H⋯O, O—H⋯N and N—H⋯O hydrogen bonds. ne of the five lattice water molecules is equally disordered around an inversion centre and shows half-occupancy.

## Related literature

For the strontium 1*H*-benzimidazole-5,6-dicarboxyl­ate deriv­ative, see: Song *et al.* (2009 ▶).
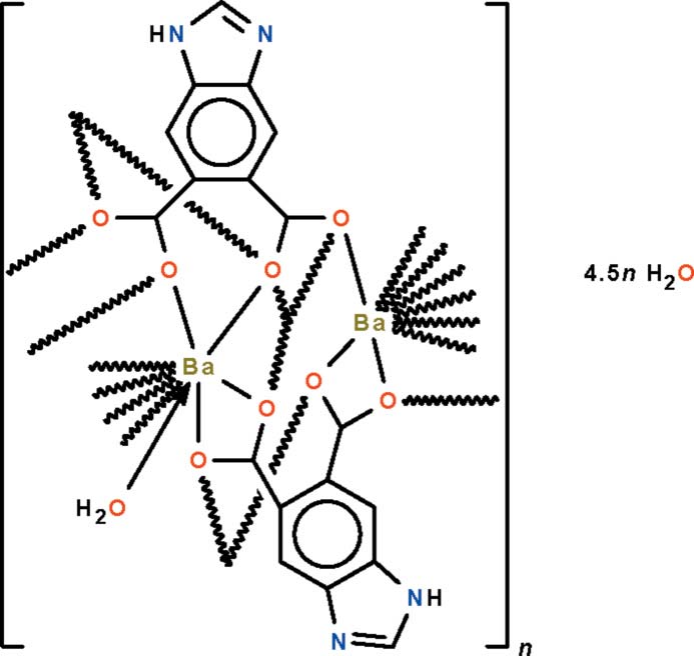


## Experimental

### 

#### Crystal data

[Ba_2_(C_9_H_4_N_2_O_4_)_2_(H_2_O)]·4.5H_2_O
*M* *_r_* = 782.05
Triclinic, 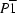

*a* = 6.9331 (4) Å
*b* = 9.5950 (4) Å
*c* = 18.0179 (7) Å
α = 103.186 (1)°
β = 92.068 (2)°
γ = 93.032 (2)°
*V* = 1163.94 (9) Å^3^
*Z* = 2
Mo *K*α radiation
μ = 3.44 mm^−1^
*T* = 293 K
0.33 × 0.24 × 0.21 mm

#### Data collection

Rigaku R-AXIS RAPID diffractometer
Absorption correction: multi-scan (*ABSCOR*; Higashi, 1995 ▶) *T*_min_ = 0.396, *T*_max_ = 0.532
11398 measured reflections
5246 independent reflections
4749 reflections with *I* > 2σ(*I*)
*R*_int_ = 0.025

#### Refinement

*R*[*F* ^2^ > 2σ(*F* ^2^)] = 0.028
*wR*(*F* ^2^) = 0.074
*S* = 1.07
5246 reflections
343 parameters
30 restraints
H-atom parameters constrained
Δρ_max_ = 1.54 e Å^−3^
Δρ_min_ = −0.68 e Å^−3^

### 

Data collection: *RAPID-AUTO* (Rigaku Corporation, 1998 ▶); cell refinement: *RAPID-AUTO*; data reduction: *CrystalStructure* (Rigaku/MSC, 2002 ▶); program(s) used to solve structure: *SHELXS97* (Sheldrick, 2008 ▶); program(s) used to refine structure: *SHELXL97* (Sheldrick, 2008 ▶); molecular graphics: *X-SEED* (Barbour, 2001 ▶); software used to prepare material for publication: *publCIF* (Westrip, 2010 ▶).

## Supplementary Material

Crystal structure: contains datablocks global, I. DOI: 10.1107/S160053681101590X/jh2280sup1.cif

Structure factors: contains datablocks I. DOI: 10.1107/S160053681101590X/jh2280Isup2.hkl

Additional supplementary materials:  crystallographic information; 3D view; checkCIF report

## Figures and Tables

**Table 1:** Hydrogen-bond geometry (Å, °)

*D*—H⋯*A*	*D*—H	H⋯*A*	*D*⋯*A*	*D*—H⋯*A*
O1*w*—H11⋯O4*w*^i^	0.84	1.66	2.49 (1)	170
O1*w*—H12⋯O2*w*^ii^	0.84	1.88	2.60 (1)	143
O2*w*—H21⋯N2	0.84	2.00	2.83 (1)	168
O2*w*—H22⋯N2^iii^	0.84	2.28	2.83 (1)	124
O3*w*—H31⋯N3	0.84	2.34	2.95 (1)	129
O3*w*—H32⋯O6*w*^iv^	0.84	1.85	2.68 (1)	174
O4*w*—H41⋯O3*w*^v^	0.84	2.03	2.84 (2)	161
O5*w*—H51⋯O1*w*^v^	0.84	2.31	2.75 (1)	113
O6*w*—H61⋯O4^vi^	0.84	1.99	2.76 (1)	152
O6*w*—H62⋯O8	0.84	2.09	2.86 (1)	152
N1—H1⋯O1*w*	0.88	1.93	2.80 (1)	168
N4—H4⋯O4*w*	0.88	1.99	2.86 (1)	167

Symmetry codes: (i) 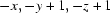; (ii) 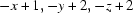; (iii) 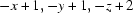; (iv) 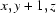; (v) 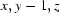; (vi) 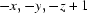.

**Fig. 1.**
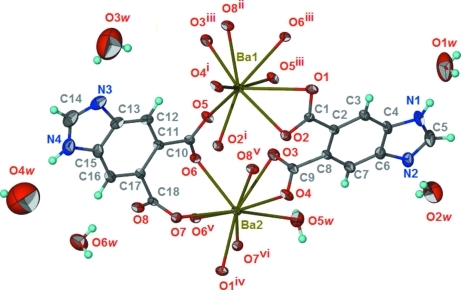
Thermal ellipsoid plot (Barbour, 2001) of a portion of the layer structure of Ba2(H2O)(C9H4NO4)2.4.5H2O at the 50% probability level; hydrogen atoms are drawn as spheres of arbitrary radius. Symmetry code: (i) -x, -y + 1, -z + 1; (ii) -x + 1, -y + 1, -z + 1; (iii) x, y + 1, z; (iv) x, y - 1, z; (v) -x + 1, -y, -z + 1; (vi) -x, -y, -z + 1.

## supplementary crystallographic information

### Comment

The 1*H*-benzimidazole-5,6-dicarboxylate dianion affords a large number of metal salts; most of the crystal structure studies involve rare earth metals. The crystal structures of only few main group derivatives have been reported. The Sr^II^ derivative exists as a monohydrate; the metal atom is also coordinated by two water ligands in an eight-coordinate square-antiprismatic geometry. The dianion functions in a \m4 bridging mode (Song *et al.*, 2009). The Ba^II^ analog has 4.5 lattice water molecules (Scheme I). Polymeric Ba_2_(H_2_O)(C_9_H_4_NO_4_)_2_^.^4.5H_2_O adopts a layer structure in which adjacent Ba^II^ atoms are connected by two benzimidazole-5,6-dicarboxylate dianions, one functioning in a µ_4_ bridging mode and the other in a µ_5_ bridging mode. The Ba atom having water in its coordination environment as well as the Ba atom without water exist in a nine-coordinate polyhedron of O atom; the geometry is undefined. Lattice water molecules occupy the space between layers, and interact with the layers through O–H···O and N–H···O hydrogen bonds to generate a three-dimensional network (Table 1).

### Experimental

Barium chloride (0.0416 g, 0.20 mmol) and 1*H*-benzimidazole-5,6-dicarboxylic acid (0.0412 g, 0.20 mmol) were placed in water (35 ml); the reactants dissolved upon addition of several drops of dilute sodium hydroxide to a pH of 7. The solution was set aside for the growth of crystals over several weeks; yield 60% based on Ba.

### Refinement

Hydrogen atoms were placed in calculated positions (C–H 0.93 Å) and were included in the refinement in the riding model approximation, with *U*(H) set to 1.2*U*_eq_(C). The amino and water H atoms were placed in chemically sensible positions on the basis of hydrogen bonding interactions (O–H 0.84 Å and N–H 0.88 Å); their temperature factors were tied by a factor of 1.2–1.5 times. Positioning the H atoms lead to H12···H21 and H31···H42 distances of about 2 Å, which are regarded as being acceptable. The O5w molecule then forms only one hydrogen bond. Positioning the second H atom elsewhere led to too short interactions.

On this basis, the N1 and N4 atoms are atoms having a hydrogen connected to them whereas the N2 and N3 atoms do not.

One of the water molecules (O5w) is disordered about a center-of-inversion.

The anisotropic temperature factors of the free water molecules were restrained to be nearly isotropic.

The final difference Fourier map had peaks in the vicinity of both Ba atoms.

### Crystal data

**Table:**

[Ba_2_(C_9_H_4_N_2_O_4_)_2_(H_2_O)]·4.5H_2_O	*Z* = 2
*M**_r_* = 782.05	*F*(000) = 750
Triclinic, *P*1	*D*_x_ = 2.231 Mg m^−^^3^
Hall symbol: -P 1	Mo *K*α radiation, λ = 0.71073 Å
*a* = 6.9331 (4) Å	Cell parameters from 10107 reflections
*b* = 9.5950 (4) Å	θ = 3.1–27.5°
*c* = 18.0179 (7) Å	µ = 3.44 mm^−^^1^
α = 103.186 (1)°	*T* = 293 K
β = 92.068 (2)°	Prism, colorless
γ = 93.032 (2)°	0.33 × 0.24 × 0.21 mm
*V* = 1163.94 (9) Å^3^	

### Data collection

**Table:**

Rigaku R-AXIS RAPID diffractometer	5246 independent reflections
Radiation source: fine-focus sealed tube	4749 reflections with *I* > 2σ(*I*)
graphite	*R*_int_ = 0.025
Detector resolution: 10.000 pixels mm^-1^	θ_max_ = 27.5°, θ_min_ = 3.1°
ω scans	*h* = −8→8
Absorption correction: multi-scan (*ABSCOR*; Higashi, 1995)	*k* = −11→12
*T*_min_ = 0.396, *T*_max_ = 0.532	*l* = −23→23
11398 measured reflections	

### Refinement

**Table:**

Refinement on *F*^2^	Primary atom site location: structure-invariant direct methods
Least-squares matrix: full	Secondary atom site location: difference Fourier map
*R*[*F*^2^ > 2σ(*F*^2^)] = 0.028	Hydrogen site location: inferred from neighbouring sites
*wR*(*F*^2^) = 0.074	H-atom parameters constrained
*S* = 1.07	*w* = 1/[σ^2^(*F*_o_^2^) + (0.0388*P*)^2^ + 1.7294*P*] where *P* = (*F*_o_^2^ + 2*F*_c_^2^)/3
5246 reflections	(Δ/σ)_max_ = 0.001
343 parameters	Δρ_max_ = 1.54 e Å^−^^3^
30 restraints	Δρ_min_ = −0.68 e Å^−^^3^

### Fractional atomic coordinates and isotropic or equivalent isotropic displacement parameters (Å^2^)

**Table:**

	*x*	*y*	*z*	*U*_iso_*/*U*_eq_	Occ. (<1)
Ba1	0.26276 (3)	0.610140 (19)	0.450910 (11)	0.02043 (7)	
Ba2	0.26062 (3)	0.078630 (18)	0.581428 (11)	0.01842 (7)	
O1	0.1686 (4)	0.8140 (3)	0.60189 (15)	0.0296 (5)	
O2	0.0501 (4)	0.5894 (3)	0.57721 (14)	0.0267 (5)	
O3	0.3591 (4)	0.3818 (3)	0.61724 (16)	0.0335 (6)	
O4	0.0593 (4)	0.3350 (3)	0.64668 (16)	0.0325 (6)	
O5	0.4310 (4)	0.3577 (2)	0.43836 (15)	0.0282 (5)	
O6	0.4612 (3)	0.1417 (2)	0.46119 (14)	0.0228 (5)	
O7	0.1291 (3)	−0.0557 (3)	0.43544 (14)	0.0259 (5)	
O8	0.3566 (3)	−0.1786 (3)	0.37175 (15)	0.0262 (5)	
O1w	0.3097 (12)	1.1921 (6)	0.8946 (3)	0.128 (2)	
H11	0.2025	1.2235	0.9081	0.192*	
H12	0.3998	1.2440	0.9213	0.192*	
O2w	0.5133 (16)	0.5633 (9)	1.0382 (5)	0.083 (3)	0.50
H21	0.4591	0.5883	1.0013	0.124*	0.50
H22	0.5884	0.4986	1.0218	0.124*	0.50
O3w	0.2112 (15)	0.4994 (10)	0.1075 (5)	0.177 (4)	
H31	0.1689	0.4135	0.0922	0.266*	
H32	0.2282	0.5210	0.1552	0.266*	
O4w	−0.0138 (16)	−0.3037 (13)	0.0517 (7)	0.225 (5)	
H41	0.0288	−0.3661	0.0729	0.337*	
H42	−0.0152	−0.3286	0.0039	0.337*	
O5w	0.3529 (5)	0.1624 (4)	0.74063 (18)	0.0466 (8)	
H51	0.2820	0.1137	0.7634	0.070*	
H52	0.4694	0.1486	0.7492	0.070*	
O6w	0.2361 (6)	−0.4351 (4)	0.2606 (3)	0.0666 (11)	
H61	0.1264	−0.4298	0.2790	0.100*	
H62	0.3069	−0.3650	0.2855	0.100*	
N1	0.3285 (6)	0.9029 (4)	0.8973 (2)	0.0397 (8)	
H1	0.3280	0.9966	0.9036	0.048*	
N2	0.3607 (6)	0.6883 (4)	0.9229 (2)	0.0424 (9)	
N3	0.2152 (6)	0.2189 (5)	0.1479 (2)	0.0434 (9)	
N4	0.1238 (6)	−0.0140 (5)	0.1170 (2)	0.0473 (10)	
H4	0.0825	−0.0992	0.0897	0.057*	
C1	0.1336 (4)	0.6990 (3)	0.62169 (19)	0.0198 (6)	
C2	0.1978 (5)	0.6878 (3)	0.70029 (19)	0.0199 (6)	
C3	0.2313 (5)	0.8127 (4)	0.7571 (2)	0.0250 (7)	
H3A	0.2173	0.9023	0.7467	0.030*	
C4	0.2863 (6)	0.7997 (4)	0.8300 (2)	0.0290 (7)	
C5	0.3694 (7)	0.8313 (5)	0.9496 (2)	0.0459 (11)	
H5	0.4014	0.8757	1.0004	0.055*	
C6	0.3078 (6)	0.6659 (4)	0.8458 (2)	0.0305 (8)	
C7	0.2809 (6)	0.5397 (4)	0.7887 (2)	0.0294 (8)	
H7	0.2983	0.4506	0.7992	0.035*	
C8	0.2277 (5)	0.5522 (3)	0.71628 (19)	0.0208 (6)	
C9	0.2134 (5)	0.4149 (3)	0.6539 (2)	0.0241 (7)	
C10	0.4047 (4)	0.2236 (3)	0.41961 (19)	0.0198 (6)	
C11	0.3099 (5)	0.1562 (3)	0.34209 (19)	0.0201 (6)	
C12	0.3057 (5)	0.2372 (4)	0.2876 (2)	0.0271 (7)	
H12A	0.3482	0.3337	0.2999	0.033*	
C13	0.2370 (5)	0.1713 (4)	0.2147 (2)	0.0306 (8)	
C14	0.1482 (8)	0.1045 (6)	0.0937 (3)	0.0517 (12)	
H14	0.1213	0.1093	0.0434	0.062*	
C15	0.1772 (6)	0.0235 (4)	0.1942 (2)	0.0312 (8)	
C16	0.1771 (5)	−0.0567 (4)	0.2494 (2)	0.0285 (7)	
H16	0.1342	−0.1530	0.2371	0.034*	
C17	0.2418 (5)	0.0098 (3)	0.32264 (19)	0.0206 (6)	
C18	0.2422 (5)	−0.0791 (3)	0.38180 (19)	0.0201 (6)	

### Atomic displacement parameters (Å^2^)

**Table:**

	*U*^11^	*U*^22^	*U*^33^	*U*^12^	*U*^13^	*U*^23^
Ba1	0.01885 (11)	0.01795 (11)	0.02441 (11)	−0.00112 (7)	−0.00104 (7)	0.00554 (8)
Ba2	0.01508 (11)	0.01541 (10)	0.02374 (11)	−0.00021 (7)	0.00126 (7)	0.00261 (8)
O1	0.0343 (14)	0.0228 (12)	0.0336 (14)	−0.0030 (10)	0.0009 (11)	0.0118 (11)
O2	0.0294 (13)	0.0237 (12)	0.0247 (12)	−0.0051 (10)	−0.0022 (10)	0.0027 (10)
O3	0.0334 (15)	0.0224 (12)	0.0429 (16)	0.0048 (10)	0.0116 (12)	0.0014 (11)
O4	0.0310 (14)	0.0199 (12)	0.0433 (15)	−0.0043 (10)	−0.0035 (11)	0.0024 (11)
O5	0.0296 (14)	0.0170 (11)	0.0360 (14)	0.0010 (10)	−0.0055 (11)	0.0032 (10)
O6	0.0206 (12)	0.0219 (11)	0.0260 (12)	−0.0023 (9)	−0.0012 (9)	0.0066 (10)
O7	0.0189 (12)	0.0325 (13)	0.0245 (12)	−0.0055 (10)	0.0009 (9)	0.0042 (10)
O8	0.0210 (12)	0.0212 (11)	0.0377 (14)	0.0022 (9)	0.0004 (10)	0.0096 (11)
O1w	0.250 (7)	0.084 (3)	0.052 (3)	0.056 (4)	−0.013 (4)	0.010 (3)
O2w	0.115 (7)	0.066 (5)	0.070 (5)	0.018 (5)	−0.021 (5)	0.024 (4)
O3w	0.222 (8)	0.179 (7)	0.139 (6)	0.058 (6)	0.037 (6)	0.036 (5)
O4w	0.220 (9)	0.245 (8)	0.216 (9)	0.008 (7)	0.022 (7)	0.067 (7)
O5w	0.049 (2)	0.0500 (18)	0.0395 (17)	0.0039 (15)	0.0036 (14)	0.0080 (15)
O6w	0.058 (2)	0.046 (2)	0.089 (3)	0.0012 (17)	0.014 (2)	0.002 (2)
N1	0.051 (2)	0.0288 (16)	0.0326 (17)	0.0046 (15)	−0.0052 (15)	−0.0057 (14)
N2	0.059 (2)	0.042 (2)	0.0234 (16)	−0.0016 (17)	−0.0061 (15)	0.0048 (15)
N3	0.039 (2)	0.062 (2)	0.0366 (19)	0.0035 (18)	−0.0018 (15)	0.0271 (19)
N4	0.052 (2)	0.061 (2)	0.0265 (17)	0.0011 (19)	−0.0085 (16)	0.0065 (18)
C1	0.0138 (14)	0.0201 (15)	0.0252 (16)	0.0012 (11)	0.0034 (12)	0.0045 (13)
C2	0.0161 (15)	0.0184 (14)	0.0244 (16)	0.0021 (11)	0.0013 (12)	0.0031 (13)
C3	0.0246 (17)	0.0189 (15)	0.0304 (18)	0.0025 (13)	0.0012 (14)	0.0028 (14)
C4	0.0298 (19)	0.0263 (17)	0.0259 (17)	0.0017 (14)	−0.0002 (14)	−0.0044 (14)
C5	0.059 (3)	0.049 (3)	0.0234 (19)	0.002 (2)	−0.0075 (18)	−0.0030 (18)
C6	0.035 (2)	0.0309 (18)	0.0237 (17)	−0.0003 (15)	−0.0015 (14)	0.0036 (15)
C7	0.037 (2)	0.0222 (16)	0.0284 (18)	0.0018 (14)	−0.0023 (15)	0.0061 (14)
C8	0.0197 (16)	0.0163 (14)	0.0248 (16)	−0.0008 (12)	0.0001 (12)	0.0021 (13)
C9	0.0289 (18)	0.0157 (14)	0.0276 (17)	0.0026 (13)	−0.0018 (13)	0.0052 (13)
C10	0.0135 (14)	0.0200 (14)	0.0258 (16)	0.0013 (11)	0.0014 (12)	0.0048 (13)
C11	0.0155 (15)	0.0179 (14)	0.0272 (16)	0.0033 (11)	0.0017 (12)	0.0051 (13)
C12	0.0228 (17)	0.0273 (17)	0.0342 (19)	0.0011 (13)	0.0021 (14)	0.0128 (15)
C13	0.0247 (18)	0.041 (2)	0.0309 (19)	0.0055 (15)	0.0008 (14)	0.0178 (17)
C14	0.049 (3)	0.079 (4)	0.030 (2)	0.004 (3)	−0.0050 (19)	0.022 (2)
C15	0.0285 (19)	0.038 (2)	0.0248 (17)	0.0024 (15)	−0.0037 (14)	0.0022 (16)
C16	0.0274 (18)	0.0265 (17)	0.0292 (18)	0.0013 (14)	−0.0015 (14)	0.0019 (15)
C17	0.0166 (15)	0.0202 (15)	0.0248 (16)	0.0026 (12)	0.0006 (12)	0.0044 (13)
C18	0.0157 (15)	0.0176 (14)	0.0258 (16)	−0.0047 (11)	−0.0032 (12)	0.0044 (13)

### Geometric parameters (Å, °)

**Table:**

Ba1—O1	3.080 (3)	O3w—H31	0.8400
Ba1—O2	2.795 (2)	O3w—H32	0.8400
Ba1—O2^i^	2.769 (2)	O4w—H41	0.8401
Ba1—O3^ii^	2.940 (3)	O4w—H42	0.8400
Ba1—O4^i^	2.936 (3)	O5w—H51	0.8401
Ba1—O5	2.711 (2)	O5w—H52	0.8400
Ba1—O5^ii^	2.816 (3)	O6w—H61	0.8401
Ba1—O6^ii^	3.069 (2)	O6w—H62	0.8400
Ba1—O8^iii^	2.795 (2)	N1—C5	1.318 (6)
Ba2—O1^iv^	2.695 (2)	N1—C4	1.389 (5)
Ba2—O3	2.871 (3)	N1—H1	0.8800
Ba2—O4	2.923 (3)	N2—C5	1.343 (6)
Ba2—O6	2.778 (2)	N2—C6	1.389 (5)
Ba2—O6^v^	2.928 (2)	N3—C14	1.342 (7)
Ba2—O7^vi^	2.700 (2)	N3—C13	1.387 (5)
Ba2—O7	2.751 (2)	N4—C14	1.304 (7)
Ba2—O8^v^	2.806 (2)	N4—C15	1.386 (5)
Ba2—O5w	2.836 (3)	N4—H4	0.8800
O1—C1	1.249 (4)	C1—C2	1.498 (5)
O1—Ba2^iii^	2.695 (2)	C2—C3	1.390 (5)
O2—C1	1.266 (4)	C2—C8	1.419 (4)
O2—Ba1^i^	2.769 (2)	C3—C4	1.389 (5)
O3—C9	1.242 (4)	C3—H3A	0.9300
O3—Ba1^ii^	2.940 (3)	C4—C6	1.392 (5)
O4—C9	1.266 (4)	C5—H5	0.9300
O4—Ba1^i^	2.936 (3)	C6—C7	1.398 (5)
O5—C10	1.255 (4)	C7—C8	1.376 (5)
O5—Ba1^ii^	2.816 (3)	C7—H7	0.9300
O6—C10	1.270 (4)	C8—C9	1.521 (5)
O6—Ba2^v^	2.928 (2)	C10—C11	1.509 (5)
O6—Ba1^ii^	3.069 (2)	C10—Ba1^ii^	3.290 (3)
O7—C18	1.254 (4)	C11—C12	1.384 (5)
O7—Ba2^vi^	2.700 (2)	C11—C17	1.419 (4)
O8—C18	1.259 (4)	C12—C13	1.377 (5)
O8—Ba1^iv^	2.795 (2)	C12—H12A	0.9300
O8—Ba2^v^	2.806 (2)	C13—C15	1.417 (6)
O1w—H11	0.8400	C14—H14	0.9300
O1w—H12	0.8400	C15—C16	1.388 (6)
O2w—O2w^vii^	1.613 (18)	C16—C17	1.377 (5)
O2w—H21	0.8400	C16—H16	0.9300
O2w—H22	0.8400	C17—C18	1.510 (4)
			
O5—Ba1—O2^i^	77.01 (8)	C10—O6—Ba2	124.7 (2)
O5—Ba1—O2	95.97 (8)	C10—O6—Ba2^v^	125.3 (2)
O2^i^—Ba1—O2	64.13 (8)	Ba2—O6—Ba2^v^	107.25 (7)
O5—Ba1—O8^iii^	126.34 (8)	C10—O6—Ba1^ii^	88.47 (18)
O2^i^—Ba1—O8^iii^	127.94 (7)	Ba2—O6—Ba1^ii^	99.88 (7)
O2—Ba1—O8^iii^	136.76 (7)	Ba2^v^—O6—Ba1^ii^	99.38 (7)
O5—Ba1—O5^ii^	70.23 (8)	C18—O7—Ba2^vi^	125.1 (2)
O2^i^—Ba1—O5^ii^	128.53 (7)	C18—O7—Ba2	121.1 (2)
O2—Ba1—O5^ii^	80.81 (8)	Ba2^vi^—O7—Ba2	112.68 (8)
O8^iii^—Ba1—O5^ii^	103.49 (7)	C18—O8—Ba1^iv^	113.3 (2)
O5—Ba1—O4^i^	126.10 (7)	C18—O8—Ba2^v^	112.42 (19)
O2^i^—Ba1—O4^i^	63.11 (7)	Ba1^iv^—O8—Ba2^v^	106.20 (8)
O2—Ba1—O4^i^	97.43 (8)	H11—O1w—H12	110.0
O8^iii^—Ba1—O4^i^	66.63 (7)	O2w^vii^—O2w—H21	66.4
O5^ii^—Ba1—O4^i^	163.60 (7)	O2w^vii^—O2w—H22	51.6
O5—Ba1—O3^ii^	68.84 (7)	H21—O2w—H22	109.5
O2^i^—Ba1—O3^ii^	132.66 (7)	H31—O3w—H32	110.7
O2—Ba1—O3^ii^	148.68 (8)	H41—O4w—H42	112.7
O8^iii^—Ba1—O3^ii^	60.17 (7)	Ba2—O5w—H51	109.4
O5^ii^—Ba1—O3^ii^	68.41 (8)	Ba2—O5w—H52	109.5
O4^i^—Ba1—O3^ii^	113.72 (8)	H51—O5w—H52	109.5
O5—Ba1—O6^ii^	110.03 (7)	H61—O6w—H62	107.7
O2^i^—Ba1—O6^ii^	158.74 (6)	C5—N1—C4	105.7 (4)
O2—Ba1—O6^ii^	94.83 (7)	C5—N1—H1	127.1
O8^iii^—Ba1—O6^ii^	64.97 (7)	C4—N1—H1	127.1
O5^ii^—Ba1—O6^ii^	44.11 (6)	C5—N2—C6	105.2 (4)
O4^i^—Ba1—O6^ii^	120.42 (7)	C14—N3—C13	106.3 (4)
O3^ii^—Ba1—O6^ii^	67.22 (7)	C14—N4—C15	105.0 (4)
O5—Ba1—O1	125.39 (7)	C14—N4—H4	127.5
O2^i^—Ba1—O1	103.10 (7)	C15—N4—H4	127.5
O2—Ba1—O1	43.78 (6)	O1—C1—O2	122.6 (3)
O8^iii^—Ba1—O1	97.04 (7)	O1—C1—C2	119.2 (3)
O5^ii^—Ba1—O1	68.40 (7)	O2—C1—C2	118.2 (3)
O4^i^—Ba1—O1	98.98 (7)	C3—C2—C8	120.4 (3)
O3^ii^—Ba1—O1	123.17 (7)	C3—C2—C1	118.9 (3)
O6^ii^—Ba1—O1	56.15 (6)	C8—C2—C1	120.7 (3)
O1^iv^—Ba2—O7^vi^	76.72 (8)	C4—C3—C2	118.0 (3)
O1^iv^—Ba2—O7	80.25 (8)	C4—C3—H3A	121.0
O7^vi^—Ba2—O7	67.32 (8)	C2—C3—H3A	121.0
O1^iv^—Ba2—O6	125.88 (8)	C3—C4—N1	131.1 (4)
O7^vi^—Ba2—O6	116.89 (7)	C3—C4—C6	121.1 (3)
O7—Ba2—O6	62.22 (7)	N1—C4—C6	107.7 (3)
O1^iv^—Ba2—O8^v^	113.68 (8)	N1—C5—N2	113.9 (4)
O7^vi^—Ba2—O8^v^	163.12 (8)	N1—C5—H5	123.1
O7—Ba2—O8^v^	126.06 (7)	N2—C5—H5	123.1
O6—Ba2—O8^v^	68.87 (7)	N2—C6—C4	107.4 (3)
O1^iv^—Ba2—O5w	87.21 (9)	N2—C6—C7	131.1 (4)
O7^vi^—Ba2—O5w	106.45 (9)	C4—C6—C7	121.4 (3)
O7—Ba2—O5w	166.97 (8)	C8—C7—C6	117.6 (3)
O6—Ba2—O5w	129.48 (9)	C8—C7—H7	121.2
O8^v^—Ba2—O5w	62.57 (9)	C6—C7—H7	121.2
O1^iv^—Ba2—O3	159.72 (8)	C7—C8—C2	121.4 (3)
O7^vi^—Ba2—O3	104.58 (8)	C7—C8—C9	116.6 (3)
O7—Ba2—O3	119.27 (8)	C2—C8—C9	121.9 (3)
O6—Ba2—O3	72.16 (7)	O3—C9—O4	123.7 (3)
O8^v^—Ba2—O3	60.90 (8)	O3—C9—C8	118.1 (3)
O5w—Ba2—O3	72.92 (9)	O4—C9—C8	118.0 (3)
O1^iv^—Ba2—O4	124.90 (8)	O5—C10—O6	123.3 (3)
O7^vi^—Ba2—O4	63.37 (7)	O5—C10—C11	118.2 (3)
O7—Ba2—O4	113.76 (8)	O6—C10—C11	118.4 (3)
O6—Ba2—O4	106.09 (7)	O5—C10—Ba1^ii^	57.20 (18)
O8^v^—Ba2—O4	100.03 (7)	O6—C10—Ba1^ii^	68.83 (18)
O5w—Ba2—O4	70.81 (9)	C11—C10—Ba1^ii^	159.4 (2)
O3—Ba2—O4	44.87 (7)	C12—C11—C17	120.1 (3)
O1^iv^—Ba2—O6^v^	61.79 (7)	C12—C11—C10	118.3 (3)
O7^vi^—Ba2—O6^v^	129.53 (7)	C17—C11—C10	121.3 (3)
O7—Ba2—O6^v^	77.96 (7)	C13—C12—C11	118.3 (3)
O6—Ba2—O6^v^	72.75 (7)	C13—C12—H12A	120.9
O8^v^—Ba2—O6^v^	66.84 (7)	C11—C12—H12A	120.9
O5w—Ba2—O6^v^	99.26 (8)	C12—C13—N3	133.1 (4)
O3—Ba2—O6^v^	124.42 (7)	C12—C13—C15	121.7 (3)
O4—Ba2—O6^v^	166.54 (7)	N3—C13—C15	105.1 (4)
C1—O1—Ba2^iii^	171.4 (2)	N4—C14—N3	114.6 (4)
C1—O1—Ba1	82.76 (19)	N4—C14—H14	122.7
Ba2^iii^—O1—Ba1	104.56 (8)	N3—C14—H14	122.7
C1—O2—Ba1^i^	147.4 (2)	N4—C15—C16	131.3 (4)
C1—O2—Ba1	95.42 (19)	N4—C15—C13	108.9 (4)
Ba1^i^—O2—Ba1	115.87 (8)	C16—C15—C13	119.9 (3)
C9—O3—Ba2	95.3 (2)	C17—C16—C15	118.4 (3)
C9—O3—Ba1^ii^	163.6 (2)	C17—C16—H16	120.8
Ba2—O3—Ba1^ii^	100.86 (8)	C15—C16—H16	120.8
C9—O4—Ba2	92.3 (2)	C16—C17—C11	121.5 (3)
C9—O4—Ba1^i^	118.6 (2)	C16—C17—C18	117.8 (3)
Ba2—O4—Ba1^i^	114.25 (9)	C11—C17—C18	120.7 (3)
C10—O5—Ba1	145.3 (2)	O7—C18—O8	123.6 (3)
C10—O5—Ba1^ii^	100.8 (2)	O7—C18—C17	120.6 (3)
Ba1—O5—Ba1^ii^	109.77 (8)	O8—C18—C17	115.8 (3)
			
O5—Ba1—O1—C1	−35.8 (2)	O3—Ba2—O7—C18	74.1 (3)
O2^i^—Ba1—O1—C1	47.5 (2)	O4—Ba2—O7—C18	124.3 (2)
O2—Ba1—O1—C1	20.40 (18)	O6^v^—Ba2—O7—C18	−48.8 (2)
O8^iii^—Ba1—O1—C1	179.1 (2)	O1^iv^—Ba2—O7—Ba2^vi^	79.59 (10)
O5^ii^—Ba1—O1—C1	−79.1 (2)	O7^vi^—Ba2—O7—Ba2^vi^	0.0
O4^i^—Ba1—O1—C1	111.8 (2)	O6—Ba2—O7—Ba2^vi^	−140.71 (12)
O3^ii^—Ba1—O1—C1	−122.06 (19)	O8^v^—Ba2—O7—Ba2^vi^	−168.15 (7)
O6^ii^—Ba1—O1—C1	−127.5 (2)	O5w—Ba2—O7—Ba2^vi^	63.6 (4)
O5—Ba1—O1—Ba2^iii^	139.56 (8)	O3—Ba2—O7—Ba2^vi^	−94.51 (10)
O2^i^—Ba1—O1—Ba2^iii^	−137.20 (9)	O4—Ba2—O7—Ba2^vi^	−44.37 (11)
O2—Ba1—O1—Ba2^iii^	−164.28 (15)	O6^v^—Ba2—O7—Ba2^vi^	142.60 (10)
O8^iii^—Ba1—O1—Ba2^iii^	−5.53 (9)	Ba1—O1—C1—O2	−39.2 (3)
O5^ii^—Ba1—O1—Ba2^iii^	96.23 (9)	Ba1—O1—C1—C2	138.9 (3)
O4^i^—Ba1—O1—Ba2^iii^	−72.88 (9)	Ba1^i^—O2—C1—O1	−120.1 (4)
O3^ii^—Ba1—O1—Ba2^iii^	53.27 (12)	Ba1—O2—C1—O1	44.0 (3)
O6^ii^—Ba1—O1—Ba2^iii^	47.87 (8)	Ba1^i^—O2—C1—C2	61.8 (5)
O5—Ba1—O2—C1	117.0 (2)	Ba1—O2—C1—C2	−134.1 (2)
O2^i^—Ba1—O2—C1	−170.5 (3)	O1—C1—C2—C3	22.3 (5)
O8^iii^—Ba1—O2—C1	−51.7 (2)	O2—C1—C2—C3	−159.5 (3)
O5^ii^—Ba1—O2—C1	48.2 (2)	O1—C1—C2—C8	−156.5 (3)
O4^i^—Ba1—O2—C1	−115.3 (2)	O2—C1—C2—C8	21.7 (5)
O3^ii^—Ba1—O2—C1	58.8 (3)	C8—C2—C3—C4	−2.7 (5)
O6^ii^—Ba1—O2—C1	6.3 (2)	C1—C2—C3—C4	178.5 (3)
O1—Ba1—O2—C1	−20.05 (18)	C2—C3—C4—N1	−179.8 (4)
O5—Ba1—O2—Ba1^i^	−72.43 (10)	C2—C3—C4—C6	0.2 (6)
O2^i^—Ba1—O2—Ba1^i^	0.0	C5—N1—C4—C3	179.1 (4)
O8^iii^—Ba1—O2—Ba1^i^	118.81 (11)	C5—N1—C4—C6	−0.9 (5)
O5^ii^—Ba1—O2—Ba1^i^	−141.24 (11)	C4—N1—C5—N2	0.8 (6)
O4^i^—Ba1—O2—Ba1^i^	55.24 (10)	C6—N2—C5—N1	−0.4 (6)
O3^ii^—Ba1—O2—Ba1^i^	−130.67 (12)	C5—N2—C6—C4	−0.3 (5)
O6^ii^—Ba1—O2—Ba1^i^	176.81 (9)	C5—N2—C6—C7	178.3 (5)
O1—Ba1—O2—Ba1^i^	150.49 (16)	C3—C4—C6—N2	−179.3 (4)
O1^iv^—Ba2—O3—C9	−57.0 (4)	N1—C4—C6—N2	0.7 (5)
O7^vi^—Ba2—O3—C9	34.1 (2)	C3—C4—C6—C7	2.0 (6)
O7—Ba2—O3—C9	106.0 (2)	N1—C4—C6—C7	−178.0 (4)
O6—Ba2—O3—C9	148.1 (2)	N2—C6—C7—C8	−179.9 (4)
O8^v^—Ba2—O3—C9	−136.6 (2)	C4—C6—C7—C8	−1.5 (6)
O5w—Ba2—O3—C9	−69.0 (2)	C6—C7—C8—C2	−1.1 (5)
O4—Ba2—O3—C9	10.7 (2)	C6—C7—C8—C9	175.7 (3)
O6^v^—Ba2—O3—C9	−158.6 (2)	C3—C2—C8—C7	3.2 (5)
O1^iv^—Ba2—O3—Ba1^ii^	120.0 (2)	C1—C2—C8—C7	−178.0 (3)
O7^vi^—Ba2—O3—Ba1^ii^	−148.87 (8)	C3—C2—C8—C9	−173.4 (3)
O7—Ba2—O3—Ba1^ii^	−76.98 (10)	C1—C2—C8—C9	5.4 (5)
O6—Ba2—O3—Ba1^ii^	−34.84 (8)	Ba2—O3—C9—O4	−21.3 (4)
O8^v^—Ba2—O3—Ba1^ii^	40.43 (8)	Ba1^ii^—O3—C9—O4	169.0 (7)
O5w—Ba2—O3—Ba1^ii^	108.05 (11)	Ba2—O3—C9—C8	152.6 (3)
O4—Ba2—O3—Ba1^ii^	−172.25 (15)	Ba1^ii^—O3—C9—C8	−17.2 (11)
O6^v^—Ba2—O3—Ba1^ii^	18.42 (12)	Ba2—O4—C9—O3	20.8 (4)
O1^iv^—Ba2—O4—C9	146.5 (2)	Ba1^i^—O4—C9—O3	−98.5 (4)
O7^vi^—Ba2—O4—C9	−165.0 (2)	Ba2—O4—C9—C8	−153.0 (3)
O7—Ba2—O4—C9	−118.8 (2)	Ba1^i^—O4—C9—C8	87.6 (3)
O6—Ba2—O4—C9	−52.6 (2)	C7—C8—C9—O3	−91.7 (4)
O8^v^—Ba2—O4—C9	18.2 (2)	C2—C8—C9—O3	85.1 (4)
O5w—Ba2—O4—C9	74.3 (2)	C7—C8—C9—O4	82.5 (4)
O3—Ba2—O4—C9	−10.4 (2)	C2—C8—C9—O4	−100.7 (4)
O6^v^—Ba2—O4—C9	30.6 (4)	Ba1—O5—C10—O6	−131.3 (3)
O1^iv^—Ba2—O4—Ba1^i^	−90.55 (11)	Ba1^ii^—O5—C10—O6	20.4 (4)
O7^vi^—Ba2—O4—Ba1^i^	−42.08 (8)	Ba1—O5—C10—C11	51.7 (5)
O7—Ba2—O4—Ba1^i^	4.13 (11)	Ba1^ii^—O5—C10—C11	−156.6 (2)
O6—Ba2—O4—Ba1^i^	70.37 (10)	Ba1—O5—C10—Ba1^ii^	−151.7 (4)
O8^v^—Ba2—O4—Ba1^i^	141.10 (9)	Ba2—O6—C10—O5	82.7 (4)
O5w—Ba2—O4—Ba1^i^	−162.74 (12)	Ba2^v^—O6—C10—O5	−118.7 (3)
O3—Ba2—O4—Ba1^i^	112.48 (14)	Ba1^ii^—O6—C10—O5	−18.3 (3)
O6^v^—Ba2—O4—Ba1^i^	153.5 (2)	Ba2—O6—C10—C11	−100.4 (3)
O2^i^—Ba1—O5—C10	10.7 (4)	Ba2^v^—O6—C10—C11	58.2 (3)
O2—Ba1—O5—C10	72.3 (4)	Ba1^ii^—O6—C10—C11	158.7 (3)
O8^iii^—Ba1—O5—C10	−117.2 (4)	Ba2—O6—C10—Ba1^ii^	100.96 (18)
O5^ii^—Ba1—O5—C10	150.3 (5)	Ba2^v^—O6—C10—Ba1^ii^	−100.42 (18)
O4^i^—Ba1—O5—C10	−31.4 (4)	O5—C10—C11—C12	17.9 (5)
O3^ii^—Ba1—O5—C10	−136.0 (4)	O6—C10—C11—C12	−159.2 (3)
O6^ii^—Ba1—O5—C10	169.7 (4)	Ba1^ii^—C10—C11—C12	−53.5 (7)
O1—Ba1—O5—C10	107.6 (4)	O5—C10—C11—C17	−167.7 (3)
O2^i^—Ba1—O5—Ba1^ii^	−139.66 (10)	O6—C10—C11—C17	15.2 (5)
O2—Ba1—O5—Ba1^ii^	−77.98 (9)	Ba1^ii^—C10—C11—C17	121.0 (5)
O8^iii^—Ba1—O5—Ba1^ii^	92.48 (11)	C17—C11—C12—C13	−1.0 (5)
O5^ii^—Ba1—O5—Ba1^ii^	0.0	C10—C11—C12—C13	173.5 (3)
O4^i^—Ba1—O5—Ba1^ii^	178.30 (8)	C11—C12—C13—N3	−179.2 (4)
O3^ii^—Ba1—O5—Ba1^ii^	73.73 (10)	C11—C12—C13—C15	−2.0 (6)
O6^ii^—Ba1—O5—Ba1^ii^	19.40 (11)	C14—N3—C13—C12	177.3 (5)
O1—Ba1—O5—Ba1^ii^	−42.68 (12)	C14—N3—C13—C15	−0.2 (5)
O1^iv^—Ba2—O6—C10	128.5 (2)	C15—N4—C14—N3	0.6 (6)
O7^vi^—Ba2—O6—C10	35.7 (3)	C13—N3—C14—N4	−0.2 (6)
O7—Ba2—O6—C10	76.6 (2)	C14—N4—C15—C16	178.2 (5)
O8^v^—Ba2—O6—C10	−126.9 (3)	C14—N4—C15—C13	−0.7 (5)
O5w—Ba2—O6—C10	−110.3 (3)	C12—C13—C15—N4	−177.3 (4)
O3—Ba2—O6—C10	−62.0 (2)	N3—C13—C15—N4	0.6 (5)
O4—Ba2—O6—C10	−32.2 (3)	C12—C13—C15—C16	3.6 (6)
O6^v^—Ba2—O6—C10	161.8 (3)	N3—C13—C15—C16	−178.5 (4)
O1^iv^—Ba2—O6—Ba2^v^	−33.33 (12)	N4—C15—C16—C17	179.1 (4)
O7^vi^—Ba2—O6—Ba2^v^	−126.14 (8)	C13—C15—C16—C17	−2.1 (6)
O7—Ba2—O6—Ba2^v^	−85.22 (9)	C15—C16—C17—C11	−0.8 (5)
O8^v^—Ba2—O6—Ba2^v^	71.25 (8)	C15—C16—C17—C18	−179.5 (3)
O5w—Ba2—O6—Ba2^v^	87.86 (12)	C12—C11—C17—C16	2.4 (5)
O3—Ba2—O6—Ba2^v^	136.20 (10)	C10—C11—C17—C16	−171.9 (3)
O4—Ba2—O6—Ba2^v^	165.99 (7)	C12—C11—C17—C18	−178.9 (3)
O6^v^—Ba2—O6—Ba2^v^	0.0	C10—C11—C17—C18	6.8 (5)
O1^iv^—Ba2—O6—Ba1^ii^	−136.47 (8)	Ba2^vi^—O7—C18—O8	−115.8 (3)
O7^vi^—Ba2—O6—Ba1^ii^	130.72 (7)	Ba2—O7—C18—O8	77.0 (4)
O7—Ba2—O6—Ba1^ii^	171.65 (10)	Ba2^vi^—O7—C18—C17	61.7 (4)
O8^v^—Ba2—O6—Ba1^ii^	−31.89 (7)	Ba2—O7—C18—C17	−105.5 (3)
O5w—Ba2—O6—Ba1^ii^	−15.28 (13)	Ba1^iv^—O8—C18—O7	17.2 (4)
O3—Ba2—O6—Ba1^ii^	33.06 (8)	Ba2^v^—O8—C18—O7	−103.3 (3)
O4—Ba2—O6—Ba1^ii^	62.86 (8)	Ba1^iv^—O8—C18—C17	−160.4 (2)
O6^v^—Ba2—O6—Ba1^ii^	−103.13 (9)	Ba2^v^—O8—C18—C17	79.1 (3)
O1^iv^—Ba2—O7—C18	−111.8 (3)	C16—C17—C18—O7	−112.0 (4)
O7^vi^—Ba2—O7—C18	168.6 (3)	C11—C17—C18—O7	69.3 (4)
O6—Ba2—O7—C18	27.9 (2)	C16—C17—C18—O8	65.7 (4)
O8^v^—Ba2—O7—C18	0.5 (3)	C11—C17—C18—O8	−113.0 (3)
O5w—Ba2—O7—C18	−127.7 (4)		

Symmetry codes: (i) −*x*, −*y*+1, −*z*+1; (ii) −*x*+1, −*y*+1, −*z*+1; (iii) *x*, *y*+1, *z*; (iv) *x*, *y*−1, *z*; (v) −*x*+1, −*y*, −*z*+1; (vi) −*x*, −*y*, −*z*+1; (vii) −*x*+1, −*y*+1, −*z*+2.

### Hydrogen-bond geometry (Å, °)

**Table:**

*D*—H···*A*	*D*—H	H···*A*	*D*···*A*	*D*—H···*A*
O1w—H11···O4w^i^	0.84	1.66	2.49 (1)	170
O1w—H12···O2w^viii^	0.84	1.88	2.60 (1)	143
O2w—H21···N2	0.84	2.00	2.83 (1)	168
O2w—H22···N2^vii^	0.84	2.28	2.83 (1)	124
O3w—H31···N3	0.84	2.34	2.95 (1)	129
O3w—H32···O6w^iii^	0.84	1.85	2.68 (1)	174
O4w—H41···O3w^iv^	0.84	2.03	2.84 (2)	161
O5w—H51···O1w^iv^	0.84	2.31	2.75 (1)	113
O6w—H61···O4^vi^	0.84	1.99	2.76 (1)	152
O6w—H62···O8	0.84	2.09	2.86 (1)	152
N1—H1···O1w	0.88	1.93	2.80 (1)	168
N4—H4···O4w	0.88	1.99	2.86 (1)	167

Symmetry codes: (i) −*x*, −*y*+1, −*z*+1; (viii) −*x*+1, −*y*+2, −*z*+2; (vii) −*x*+1, −*y*+1, −*z*+2; (iii) *x*, *y*+1, *z*; (iv) *x*, *y*−1, *z*; (vi) −*x*, −*y*, −*z*+1.
